# The emergence of human metapneumovirus G gene duplication in hospitalized patients with respiratory tract infection, India, 2016–2018

**DOI:** 10.1007/s11033-022-08092-8

**Published:** 2022-11-18

**Authors:** Preetiparna Parida, Sudheesh N, Sanjay E.R, Anitha Jagadesh, Srilatha Marate, Arunkumar Govindakaranavar

**Affiliations:** 1 Manipal Institute of Virology (MIV), Manipal Academy of Higher Education (MAHE), Manipal, Karnataka 576104 India; 2 2-49, Vaikathu, Maratithota Road, MooduAthradi, Athradi PO, Udupi, Karnataka 576107 India

**Keywords:** Human metapneumovirus, Genotyping, A2c sub-lineage, Glycoprotein (G)

## Abstract

**Background:**

Human metapneumovirus (HMPV) belongs to the family *Pneumoviridae.* It is one of the emerging respiratory viruses causing both upper and lower respiratory tract illnesses. HMPV has two genotypes: A and B. These genotypes are classified into lineage A1, A2, B1 and B2. Lineage-A2 is further classified as A2a, A2b and A2c. Similarly, B2 is classified as B2a and B2b. Studies have shown the circulation of A2b, B1 and B2 lineages in India. However, a limited amount of data is available on the current circulating genotypes of HMPV in India.

**Methods:**

Throat swab samples positive for HMPV by real-time RT- PCR, archived at Manipal Institute of Virology as a part of a hospital-based acute febrile illness surveillance study, was used from April 2016 to August 2018 by purposive sampling method. We performed the conventional reverse transcriptase-polymerase chain reaction for twenty samples targeting the G gene and then subjected them to sequencing. Phylogenetic analysis was done using MEGA X software by the Maximum Likelihood method.

**Results:**

All the twenty sequences belonged to the A2c subgroup. Phylogenetic analysis showed that strains from the study have genetic relation with circulating strains in Japan, China and Croatia. Seven out of the twenty sequences showed 180-nucleotide duplication and eleven sequences showed 111-nucleotide duplication. Two sequences did not show any duplications.

**Conclusion:**

In the current study, we report that A2c is the sub-lineage in India from April 2016 to August 2018. This study is the first retrospective study reporting the circulation of the A2c sub-lineage among adults in India with 180- and 111-nucleotide duplications in the G gene of human metapneumovirus.

## Introduction

*Human metapneumovirus* (HMPV) is a single-stranded negative-sense enveloped RNA virus under the family *Pneumoviridae* [1]. The virus was first identified in the Netherlands in 2001 and has since been detected all over the globe [2] [3]. Clinical symptoms of HMPV vary from mild upper respiratory tract infection and may progress to bronchiolitis and pneumonia [4]. The prevalence of HMPV varies from 1 to 19% in different regions in India [5]. HMPV is one of the common respiratory pathogens that cause clinical diseases, similar to the human respiratory syncytial virus (RSV) [6]. The virus genome is 13.35 kb in length and encodes eight genes which encode nine proteins such as N (nucleoprotein), P (phosphoprotein), M (matrix protein), F (fusion protein), M2 (transcription enhancer protein), SH (small hydrophobic protein), G (attachment glycoprotein) and L (RNA dependent RNA polymerase) [1]. The two major genotypes of HMPV are A and B. These two genotypes are further divided into subgroups A1, A2, B1, and B2 based on the variability of sequence in the G and F glycoproteins. Subgroup A2 is further subdivided into A2a, A2b, and A2c. G and F glycoproteins are the principal surface peplomers among the viral encoded proteins.

These two proteins are responsible for eliciting protective immunity and are antigenically significant. The high degree of nucleotide variation in the HMPV G protein accounts for the genetic variation among the genotypes. The nucleotide identity of the G gene between genotypes is about 45–53%, whereas the amino acid identity is about 22–27.6% [7]. Because of the higher genetic and antigenic diversity, the G gene is mainly used for HMPV typing and phylogenetic analysis [8]. Previous studies from India have reported the circulation of sub-lineage B1, B2, and A2b in India [4] [9]. However, the majority of the research findings are based on studies that are limited to specific geographic regions and children. Here we report the genetic variability of HMPV among hospitalized patients in ten states of India from 2016 to 2018.

## Materials and methods

### Ethics statement

The Institutional Ethical Committee, Manipal Academy of Higher Education, approved the study ((IEC No: UEC/32/2013–2014, MUEC/Renewal-08/2017). We confirm that all methods followed the relevant guidelines and regulations when samples were collected. Written consent was signed by all patients or their guardian(s) in the case of children (assent form).

### Clinical samples

**Fig. 1:**
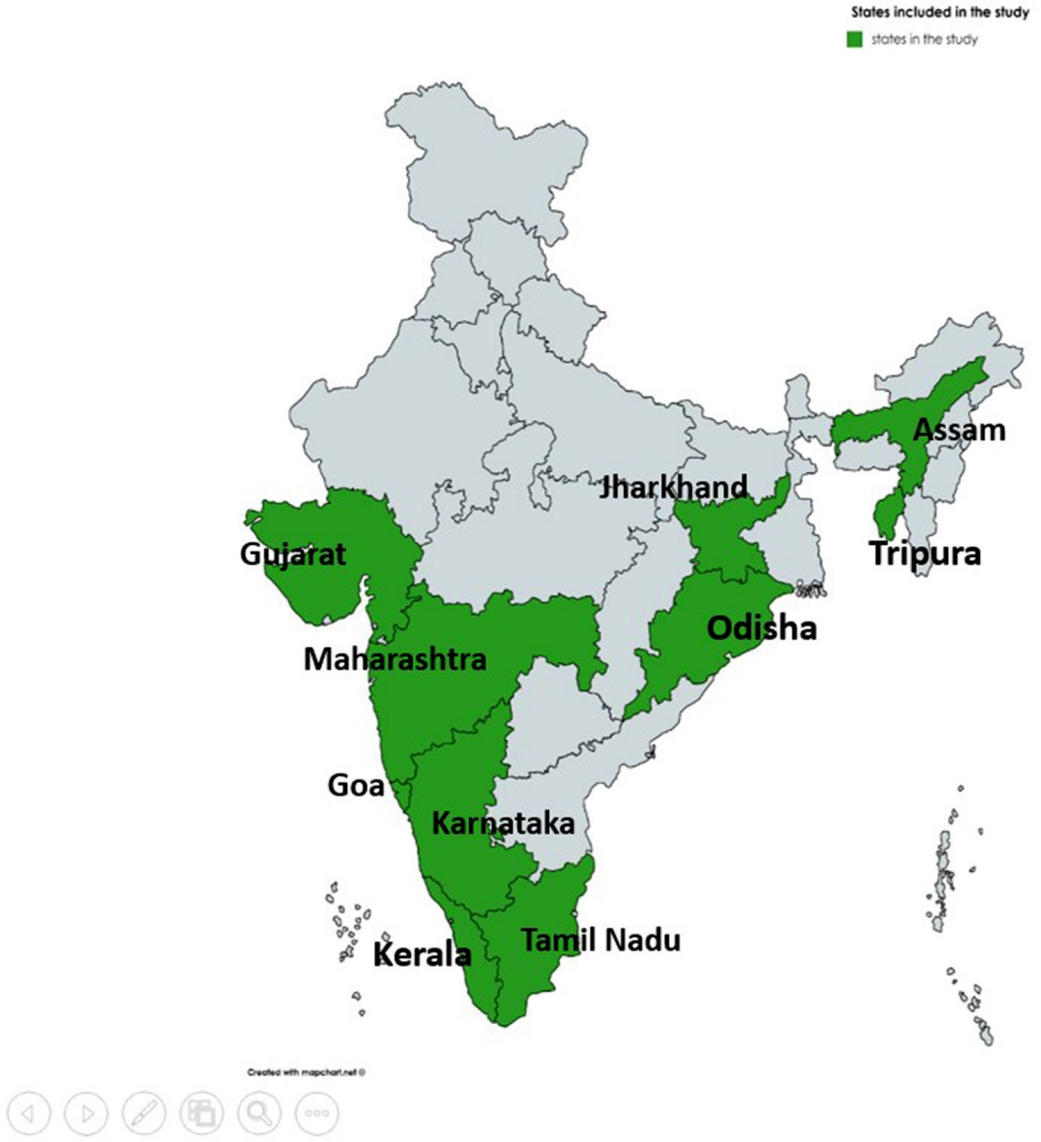
Map showing the geographical representation of study sites

Throat swab samples archived at Manipal Institute of Virology from April 2016 to September 2018 from various study sites (Fig. 1) across India, as a part of the Hospital-Based Acute Febrile Illness (AFI) surveillance study, were taken. As per the AFI operational case definition, “a sick case older than 5 years and younger than 65 years of age admitted to one of the participating hospitals with a temperature of ≥ 38 °C” were included in this study. Patients meeting the AFI operational case definition were identified and enrolled with the help of the attending physician. A study technician or nurse collected clinical history and other relevant information in a standard case recruitment form. However, children below five years of age were admitted to the hospital with a fever ≥ 38 °C and respiratory tract infections were also included.

A total of 41,008 samples were tested using FTD® Respiratory pathogen 21 Multiplex real-time PCR kit (Fast-track Diagnostics© Luxembourg S.à.r.l), of which 206 were positives for HMPV. The study population (n = 206) was categorised into age groups 0 to > 2, 2 to > 5, 5 to > 15, 15 to > 50, and 50 to > 65, following WHO guidelines for influenza surveillance [10]. The samples with a Ct value < 30 were grouped based on their geographical location (state-wise) and month of recruitment. Representative samples (n = 20) from each category with the lowest Ct value were randomly selected for conventional PCR, modified sanger sequencing and phylogenetic analysis.

### Nucleic acid extraction and reverse transcriptase-polymerase chain reaction (RTPCR)

The QIAamp Viral RNA Mini kit (catalogue no.52906, Qiagen, Hilden, Germany) was used to extract RNA from the throat swab samples. Reverse-transcription PCR (RT-PCR) was performed using primers amplifying the full-length G gene of HMPV [4] and Superscript III Platinum™ One-Step qRT-PCR Kit (catalogue. No. 1821198, Invitrogen, Thermo Fisher Scientific, Carlsbad, CA 92,008, USA). The reverse transcription was done at 50 °C for 30 min followed by initial denaturation at 95 °C for 15 min, then 40 cycles of 94 °C for 1 min, 52 °C for 1 min, and 72 °C for 1 min. A final extension was done at 72 °C for 10 min. The PCR products were subjected to agarose gel electrophoresis and observed under UV-transilluminator. The positive PCR products (n = 20) were purified using GenElute™ Gel Extraction Kit (Lot No. SLBZ2166, Sigma-Aldrich, Merck KGaA, Darmstadt, Germany). The purified products (n = 20) were sequenced using BigDye Terminator (version 3.1) cycle sequencing kit (Applied Biosystems, Foster, CA) according to the manufacturer's protocol. Purified sequences were analysed using a 3500xL Genetic analyser (Applied Biosystems, Foster City, California 94,404, USA.). All purified products were sequenced using both HMPV forward and HMPV reverse primers.

### Sequence analysis

**Fig. 2:**
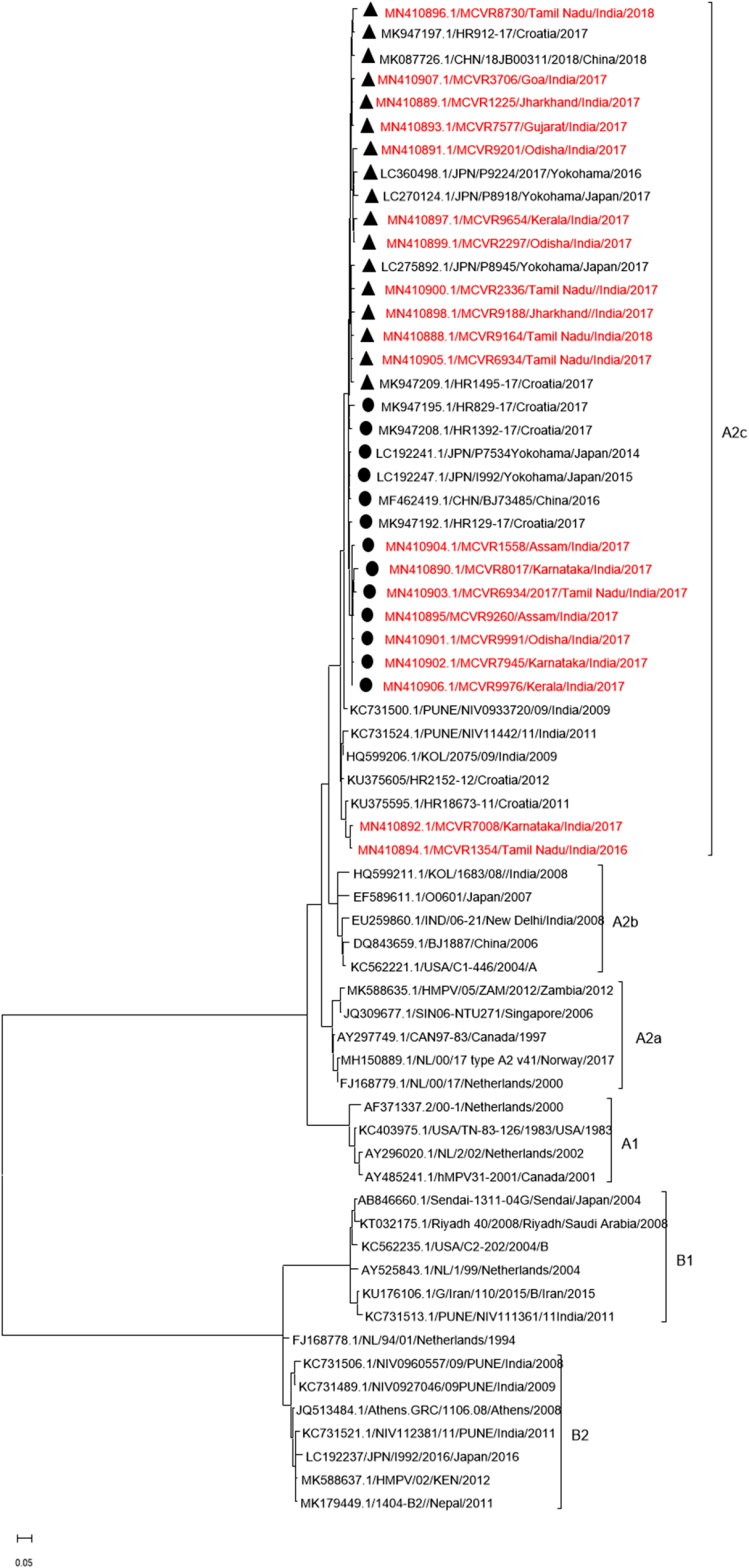
Phylogenetic tree of HMPV strains. The reference strains were obtained from the NCBI database. Sequences marked with black triangles denote 111-nucleotide duplications and black dots denote 180-nucleotide duplications in the G gene. The sequences from the current study are highlighted in red

The reference nucleotide (G gene) sequences were downloaded from the NCBI database in FASTA format (https://www.ncbi.nlm.nih.gov/) for analysis. Partial sequences (< 80%) of the G gene were excluded, and identical sequences from the same publication were removed. We analysed all the published HMPV G gene sequences from India. For comparison of our sequences with currently circulating sequences worldwide, we selected reference strains from countries that reported HMPV. The prototype strains were selected from the International Committee on Taxonomy of Viruses (ICTV) website [11], followed by multiple sequence alignment using the Clustal Omega program (https://www.ebi.ac.uk/Tools/msa/clustalo/). Sequences were analysed using Sequencher DNA analysis software (version 5.4.6). The phylogenetic analysis was performed by Maximum likelihood (ML) estimation using MEGA (Molecular Evolutionary Genetics Analysis) software Version 11, and the phylogenetic tree was constructed (Fig. 2). The best-fit nucleotide substitution model (s) for ML was chosen. Tamura-Nei model and 1000 bootstraps were applied for distance estimation. Branches with more than 75% bootstrap values were denominated as statistically significant. Basic Local Alignment Search Tool (BLAST) (http://www.ncbi.nlm.nih.gov/BLAST/) was used for sequence comparison and analysed sequences were deposited to the NCBI databank (GenBank accession numbers: MN410888 to MN410907).

## Results

### Epidemiology of HMPV

HMPV was distributed among all age groups studied. Out of 206 HMPV-positive cases, the age group 15–50 had the maximum number of positive cases, 91 (44.2%), whereas the 0–2 years had only one positive case (0.5%). The number of positive cases among other age groups such as 2–5, 5–15, and 50–65 was 26 (12.6%), 75 (36.4%), and 13 (6,3%), respectively (Table 1). The median age was 15 years (IQR = 27.75) (Table 2). Among these, 52.9% were males and 47.1% were females. The majority of the cases were presented with cough (97.5%) followed by coryza (82.5%), chills (73.3%), myalgia (64.5%), vomiting (32%), neck stiffness (27.2%), breathlessness (17.5%) and diarrhoea (4.4%). Two cases had central nervous system manifestations (0.97%). Demographic and clinical details were mentioned in Table 1.

**Table 1:** Demographic and clinical details of HMPV-positive cases

	Total no. of HMPV positives (n = 206)	
Variables	N	Percentage (%)
Age (years)		
0 to > 2 years	1	0.5
2 to > 5 years	26	12.6
5 to > 15 years	75	36.4
15 to > 50 years	91	44.2
50 to > 65 years	13	6.3
Gender		
Male	109	52.9
Female	97	47.1
Clinical features		
Cough	201	97.5
Coryza	170	82.5
Chills	151	73.3
Myalgia	133	64.5
Vomiting	66	32.0
Neck Stiffness	65	27.2
Breathlessness	36	17.5
Diarrhoea	9	4.4
Seizures	2	1.0
Altered sensorium	2	1.0

**Table 2:** Statistical analysis of HMPV positive-cases

Variables	Total no. of HMPV positives (n = 206)						
	Mean	SD	Median	IQR	Maximum	Minimum	Range
Age	20	16	15	27.8	62	1	61
Age (Male)	19.1	15.5	14	24	62	1	61
Age (Female)	21.1	16.5	16	29	60	2	58

### Co-infection with other respiratory pathogens

Twenty-eight HMPV-positive cases (13.6%) had co-infection with other pathogens such as influenza virus 7(3.4%). Leptospira 6 (2.9%), Dengue virus 4 (1.9%), Scrub Typhus 4 (1.9%), Adenovirus 2 (1%), and Enterovirus 2 (1%). Five cases (2.4%) were co-infected with other pathogens like respiratory syncytial virus, norovirus GII, parainfluenza 3, *Corynebacterium diphtheriae* and *Pseudomonas aeruginosa.*

### Phylogenetic analysis

All the HMPV sequences were clustered in the A2c sub-lineage (Fig. 2). Eleven samples had 111-nucleotide duplication and seven samples had 180-nucleotide duplication. Two samples did not show any duplication. The 111-nucleotide duplicated samples clustered with the Chinese strain (MK087726) and the Yokohama strain.

(LC270124) with 97.56% and 96.97% nucleotide identity respectively, whereas the 180-nucleotides duplicated strains clustered with strains reported in Yokohama city from 2013 to 2016. The samples without duplications clustered with Croatian (KU375595) strains and showed 96.56% nucleotide identity. It was also observed that both nucleotide duplication strains formed separate clusters. The details of samples showing nucleotide duplications in the G gene are mentioned in (Table 3).

**Table 3:** Nucleotide duplication information of HMPV genotypes obtained from the present study

Sl. no	Accession no	Genotype	Nucleotide duplication	Origin of duplication in the G gene	Position of insertion in the G gene
1	MN410888	A2c	111	420–531	532–643
2	MN410889	A2c	111	420–531	532–643
3	MN410890	A2c	180	371–550	551–731
4	MN410891	A2c	111	420–531	532–643
5	MN410892	A2c	No duplication	NA	NA
6	MN410893	A2c	111	420–531	532–643
7	MN410894	A2c	No duplication	NA	NA
8	MN410895	A2c	180	371–550	551–731
9	MN410896	A2c	111	420–531	532–643
10	MN410897	A2c	111	420–531	532–643
11	MN410898	A2c	111	420–531	532–643
12	MN410899	A2c	111	420–531	532–643
13	MN410900	A2c	111	420–531	532–643
14	MN410901	A2c	180	371–550	551–731
15	MN410902	A2c	180	371–550	551–731
16	MN410903	A2c	180	371–550	551–731
17	MN410904	A2c	180	371–550	551–731
18	MN410905	A2c	111	420–531	532–643
19	MN410906	A2c	180	371–550	551–731
20	MN410907	A2c	111	420–531	532–643

## Discussion

Earlier studies reported that the prevalence of HMPV is higher among children below two years of age with lower respiratory tract infections [12–14]. Nevertheless, subsequent studies have shown the circulation of HMPV among adults [15]. In our study, the prevalence of HMPV was found in all age categories, with a higher prevalence in the 15–50 age group, concordant with previously reported findings, emphasising the importance of HMPV in adults. A prospective cohort study of young and old adults with respiratory tract infections reported that HMPV illness rates were highest among young adults [16]. A study on HMPV from Puducherry, India, reported that the 14–30 age group had the maximum number of positive cases supporting our findings [15].

In our study, we found the co-infection of HMPV with other respiratory viruses such as influenza virus, respiratory syncytial virus, adenovirus, parainfluenza virus and enterovirus and bacteria including *Corynebacterium diphtheriae* and *Pseudomonas aeruginosa.* These results agree with previous publications which reported the co-infection of HMPV with other respiratory viral and bacterial pathogens [3]. In this study, we also detected other co-infecting viral and bacterial pathogens like norovirus GII, dengue virus, *Leptospira spp*, and Scrub typhus respectively.

Phylogenetic analysis of G genes of HMPV revealed the circulation of sub-lineage A2c in India during the study period. This study is the first retrospective study reporting the circulation of the A2c sub-lineage among adults in India. A recent study from Chennai, India, described the circulation of HMPV A2c, B1 and B2 strains among children [17]. However, our study did not find B1 and B2 sub-lineages, which were earlier reported in India [4, 18, 19]. The probable reason for missing out on these lineages might be the lower sample size chosen for sequencing. However, the sample size was small; it had a broader geographical coverage representing ten states of India.

The first case of the HMPV A2c strain was identified in Okinawa, Japan, in 2011[20]. Similar strains were reported in Malaysia [21], Croatia [22], Japan [23], Senegal [24], and China [25]. The predominant genotypes of HMPV usually change every 1–3 years due to the generation of genotype-specific antibodies in the population [26]. A2c sub-lineage of HMPV does not show any significant difference in clinical manifestation as compared to the other strains [20, 22, 27]. Our study also did not observe any distinct symptoms in any HMPV A2c-positive patients. As all of our sequenced samples belonged to the A2c sub-lineage, we could not compare symptoms between different sub-lineages.

In the present study, we observed that 7 out of 20 sequences showed 180-nucleotide duplication in the G gene of the A2c sub-lineage. Similar findings were reported in earlier studies from Barcelona, Spain, between 2014 and 2016[27], in Croatia, between 2014 and 2017 [28] and Yokohama in 2014[23]. Similarly, a 111-nucleotide duplication was also observed in 11 out of 20 sequences. Similar nucleotide duplication was previously reported in Yokohama city, Japan, between 2014 and 2016 [29], in Croatia, between 2014 and 2017 [28] and in Guangzhou, China, 2017[25]. A study conducted by Pinana et al. in Barcelona, Spain, speculated that the A2c sub-lineage might replace the A2a and A2b sub-lineages. Furthermore, they postulated that A2c strains with duplications might soon replace A2c wild type due to a better immune evasion mechanism resulting from the duplication event [27]. A similar pattern was observed in our study, where we did not find any samples positive for the A1 sub-lineage.

Eshaghi et al. reported a 72-nucleotide duplication in the ON1 genotype of subgroup A [30], and a 60-nucleotide duplication in the BA genotype of subgroup B was reported by Trento et al. [31] in the HRSV G gene. These duplications did not show any significant difference in the virulence of ON1 and BA genotypes of HRSV. However, these duplications contributed to the rapid spread of ON1 and BA genotypes of HRSV globally and made them the predominant strains in many countries [23]. A similar effect was observed for the HMPV duplication variants as well. Saikusa et al*.* reported the HMPV 180-nucleotide duplication variant, which evolved from 2011 to 2013, became the prime epidemic strain in Yokohama within three years [23]. Jagusic et al*.* reported that the recently identified HMPV duplications strains co-circulate in Croatia, which entirely replaced the former group A sub clusters in 2017 [28].

Moreover, Piñana et al. reported that the circulation of the A2c sub-lineage of HMPV is higher in adults than in children [27]. The present study results are concordant with these findings, emphasising the importance of HMPV in adults. Piñana et al. also showed that the mutated strains of HMPV had a stronger association with respiratory illnesses in adults and had less impact on children [27] No significant difference was observed in clinical symptoms of patients infected with non-duplication variants and HMPV duplication variants. Therefore, further studies are required to elucidate the role of these duplications as an evolutionary advantage for HMPV.

Our study has a few limitations, as we have included representative samples from various study sites for sequencing due to the limited availability of financial resources, making it challenging to conclude only A2c was circulating during the study period. However, continuous surveillance is necessary to see whether these 180- and 111-duplications of the G gene of HMPV have any repercussions on a wider geographic spread in future.
